# TNM Staging System in Thymoma: A Critical Appraisal?

**DOI:** 10.3390/jcm13020610

**Published:** 2024-01-21

**Authors:** Marcello Carlo Ambrogi, Vittorio Aprile, Alessandra Lenzini, Diana Bacchin, Maria Giovanna Mastromarino, Stylianos Korasidis, Marco Lucchi

**Affiliations:** 1 Division of Thoracic Surgery, Cardiac, Thoracic, and Vascular Department, University of Pisa, 56127 Pisa, Italy; marcello.ambrogi@unipi.it (M.C.A.); alessandralenzini2@gmail.com (A.L.); d.bacchin@hotmail.com (D.B.); mgmastromarino@gmail.com (M.G.M.); stylianoskorasidis@gmail.com (S.K.); marco.lucchi@unipi.it (M.L.); 2 Department of Surgical, Medical and Molecular Pathology and Critical Care Medicine, University of Pisa, 56124 Pisa, Italy

**Keywords:** thymoma, thymic epithelial tumor, staging, TNM, Masaoka-Koga

## Abstract

Thymomas are rare tumors of the anterior mediastinum with peculiar clinical and pathological features. They have been deeply analyzed by pioneer authors, who strictly linked their name to the main pathological and staging classifications. Before the latest edition of the WHO classification of thymic epithelial tumors, the history of thymoma pathological classification inherited the name of the pathologists who systematically addressed the issue, from Levine-Rosai to Muller-Hermelink. Similarly, the thymoma staging system is intimately related to the name of two surgeons, Masaoka and Koga, who historically dealt with this disease. More recently, the traditional tumor-nodes-metastasis (TNM) system has been developed for the staging of this condition, in a rational attempt to put thymomas in conformity with the other solid tumors. The efforts of the International Thymic Malignancies Interest Group (ITMIG) and the Thymic Domain of the Staging and Prognostic Factors Committee (TD-SPFC) of the International Association for the Study of Lung Cancer (IASLC) resulted in the TNM classification of thymic tumors, which have been included in the eighth edition of the American Joint Committee on Cancer’s (AJCC) Cancer Staging Manual. Herein, we report a narrative review of the evolution of the thymic epithelial tumors (TET) staging system and present a critical appraisal of the actual TNM classification compared with the historical Masaoka-Koga classification, with special focus on the proposal for the ninth edition of the TNM, expected in 2024.

## 1. Introduction

Thymomas and thymic carcinomas (thymic epithelial tumors, TETs) are rare neoplasms accounting for 0.2–1.5% of all malignancies although they represent more than 50% of all anterior mediastinal masses [1]. Both thymomas and thymic carcinomas arise from the epithelial cells of the thymus, but they are characterized by different behavior: Thymomas are usually indolent neoplasms with a tendency to increase in size with a poor infiltrative attitude, while thymic carcinomas tend to be more aggressive; in 80% of cases, there is evidence of invasion of the contiguous structures [2]. The low incidence together with the diversified biologic behavior of the thymic neoplasm has raised an active debate on histological classification and especially on the staging system in recent decades [3].

Until the late 1990s, several histopathological classifications were concurrently used, leading to great confusion among scholars. In order to address all the problems and concerns related to the diagnosis of these tumors, Suster and Moran in 1999 proposed to stratify TETs as: thymoma, atypical thymoma, and thymic carcinoma, according to the main pathological features together with clinical behavior [4]. In the same year, the jungle of varying proposals was overcome by the World Health Organization (WHO) classification, which summarized the great work of eight pathologists coordinated by Rosai and proposed an unequivocal classification based on the cytoarchitectural features of TETs [5].

According to the latter, thymomas were classified as type A thymoma, type AB thymoma, and type B thymoma, the latter separated into B1, B2, and B3 thymomas, depending on the occurrence and combination of different epithelial cells and T-lymphocytes [6].

Regarding the staging system, up to the 1970s thymic neoplasms were divided in two categories, invasive and noninvasive, according to the Bernatz classification, while in the following years Bergh et al. and Wilkins and Castleman proposed two other TET staging systems, both based exclusively on the extent and invasiveness grade of the tumor mass [7,8,9]. However, a pioneer in this field has been Akira Masaoka, who analyzed the oncological results of 96 patients treated in a single institution and proposed, in 1981, a structured staging system, which was marginally modified by Koga et al. in 1994 [10]. The Masaoka-Koga classification was mainly based on the invasiveness of the tumor through the capsule into surrounding tissues and neighboring structures [10,11] as reproduced in Table 1 [11].

The tumor, nodes, metastasis (TNM) classification is by far the most common system applied to malignant solid tumors for predicting prognoses and selecting treatment strategies. It is based on the evaluation of the tumor extent (T-factor), the nodal involvement (N-factor), and the presence of distant metastasis (M-factor). The purpose of this classification is to stratify homogeneous groups of patients with similar features and prognoses. In the case of the thymic neoplasm, however, this system has never found a broad consensus among scholars since the first attempt at TNM classification dated 1991 by Yamakawa, who proposed a T classification similar to the Masaoka staging system but distinguishing for the first time the roles of lymphogenous and hematogenous metastases [12].

As reported in Table 1, the Masaoka-Koga system was based mainly on direct tumor invasion, and contrarily to the TNM classification, it did not provide a differentiation of the eventual neighboring organs involved or a specific role for lymphatic or hematologic dissemination and pleural or pericardial droplet metastases [13]. Despite these critical issues, the Masaoka-Koga System has been employed for more than 35 years worldwide until 2017 when, for the first time, a structured TNM classification for thymic malignancies was proposed by the International Association for the Study of Lung Cancer (IASLC) and the International Thymic Malignancies Interest Group (ITMIG) on commission by the Union for International Cancer Control (UICC) and the American Joint Committee on Cancer (AJCC), which were in charge of updating and releasing the TNM classification for solid tumors [14]. According to the current TNM edition (the eighth), as reported in Table 2, the T-component is divided into 4 groups based on invasiveness or involvement level rather than size (as for other solid tumors). The N-component describes two wide categories according to the interested nodal stations level, while the M-component is divided into two distinct groups depending on the metastases site: The presence of pleural or pericardial nodules or droplet metastases is defined as M1a, while pulmonary intraparenchymal nodules and distant metastases are included in the M1b category [14,15].

When compared to the previous classifications, the TNM introduced several changes that struggled to be established during the last decade, with different papers that on a case-by-case basis validated, accepted, or denied them [15,16,17].

The aim of this narrative review is to retrace the historical evolution of the TET staging system classification, analyzing the strengths and weaknesses of the most common ones with a special focus on the next upcoming edition (the ninth) of the TNM [18].

## 2. Materials and Methods

Relevant literature up to October 2023 was searched in the PubMed and Cochrane library using as keywords: “Thymoma”, “Thymic Epithelial Tumor”, “Staging system”, “TNM”, and “Masaoka-Koga”. The search was limited to studies in English, and relevant studies were identified, screened, and reviewed by all the authors. We conducted an accurate search of all the studies focused on the Masaoka-Koga and TNM staging systems since the first proposal in 1991 by Yamakawa [12], and we selected only those studies that allowed a critical appraisal regarding this staging system, descriptor by descriptor. Unpublished material and congress abstracts were not considered.

## 3. Results

### 3.1. T-Factor

One of the most controversial aspects is the definition of the T-descriptor, which is essentially based on the local extension of the tumor and on the eventual involvement of the neighboring structures regardless of tumor size, similarly to what was proposed by Masaoka. In the eighth TNM staging for TETs (Table 2), two groups of tumors can be identified, in accordance with the organs involved (level) and the corresponding impact on prognosis: those that are potentially resectable, from T1 to T3, and those that are unresectable due to infiltration of vital organs, classified as T4.

One of the criticisms belonging to the Masaoka-Koga system that can also be encountered in the TNM classification, as reported by Detterbeck in 2018, is the challenging pre-operative evaluation of the tumor invasion by imaging, which will result in an improper staging prior to surgery and a potential loss of the opportunity for the treatment [15].

In the latest classification, the T1 category includes a large group of thymic tumors from those totally encapsulated to those extended into the adjacent mediastinal fat or involving the mediastinal pleura (Masaoka-Koga stages I, II, and III), outclassing the holy grail of the capsule and mediastinal pleura, the last barriers between the mediastinum and the pleural space, that were considered cornerstones of the previous classifications [15]. Asamura and colleagues in a retrospective study conducted to replace the Masaoka-Koga classification, reported the same 5- and 10-year survival for patients with stage I and II thymomas [19]; similarly, Okamura et al. and Nakagawa and associates found similar survival rates for stage I and II thymomas, in 273 and 130 thymomas, respectively [20,21]. In 2008, Gupta and colleagues published results of a meta-analysis regarding the prognostic importance of transcapsular invasion, and they found a similar prognosis in stage I and II thymomas; hence they suggested combining these two stages into a single one [22]. However, other authors such as Maggi [23], Lardinois [24], Roden [25], and Masaoka himself in 2010 described a significant difference between the oncological outcomes of stage I and stage II thymomas, mainly in terms of recurrence rate and disease-free survival rather than global survival, emphasizing the relevance of the transcapsular and transpleural invasion [16].

According to the Masaoka classification, the T3 category includes all thymomas invading adjacent structures with no differences between lesions with slight invasion into the pericardium, usually associated to a good prognosis, and those with aggressive invasion into the lung or great vessels, which are often considered unresectable with a consequent unfavorable prognosis [26]. The same criticisms may also be found in the TNM classification, where under the T3 category there are pooled lesions invading the phrenic nerve, whose sparing is gaining broad consensus worldwide, and tumors presenting great vessels invasion, whose prognostic implication is still unclear and highly subordinated to the surgeon’s ability and experience rather than to the disease’s biological behavior [27,28]. Yamada and colleagues in 2005 published a retrospective study using the Japanese nationwide database and described 10-year overall and disease-free survival rates as 80.2% and 51.6%, respectively, in 310 Masaoka stage III thymoma completely resected, even in those with great-vessels involvement [29]. On the other hand, Okamura et al. [30] reported that involvement of the great vessels was the only independent factor determining postoperative survival. As well, Marulli and colleagues found that the invasion of major vessels was associated with a worse prognosis even in radically treated patients [31].

Lastly, in contrast to the TNM classifications of other solid cancers, the T descriptor of the thymic neoplasms disregards the clinical or pathological size of the tumor, being based exclusively on its invasiveness and its tendency to disseminate. In the past, however, different authors described the prognostic impact of the maximal tumor diameter, albeit with different cutoff values. In 1995, Blumberg and colleagues reported better survival in patients with a thymoma smaller than 11 cm (5-year survival rate of 84% compared to 58%), while Write et al. and Lewis et al. reported a worse prognosis in patients with thymomas larger than 8 and 15 cm, respectively [32,33,34]; Ruffini et al., instead, based on data from more than 2000 patients from the European Society of Thoracic Surgeons (ESTS) database demonstrated a correlation between tumor size and both an increased risk of recurrence and incomplete resection [35].

In the view of the ninth TNM staging for TETs, the Thymic Domain of the Staging and Prognostics Factor Committee (TD-SPFC) of the International Association for the Study of Lung Cancer (IASLC) analyzed data from more than 11,000 patients, most of them surgically treated, and updated the previous classification especially regarding the T-factor as reported in Table 3 [18,36].

Proposals for the T-category can be summarized as follows: The T1 category or Level 1 structures include thymus, anterior mediastinal fat, and mediastinal pleura with a new classification of T1 into T1a (≤5 cm) and T1b (>5 cm), irrespective of mediastinal pleura invasion. The T2 category (level 2 structures) includes pericardium, lung, or phrenic nerve directly involved. The T3 category (level 3 structures) denotes direct invasion of the brachiocephalic vein, superior vena cava, chest wall, or extra pericardial pulmonary arteries and veins; and The T4 (level 4 structures) category remains the same as in the eighth edition classification, involving direct invasion of the aorta and arch vessels, intrapericardial pulmonary arteries and veins, myocardium, trachea, or esophagus (Table 3).

The latest classification recognized for the first time the significance of tumor size especially in the early stage, whereas a complete resection is advisable [18]. The authors in fact described a significantly worse overall survival in patients with a T1 thymoma greater than 5 cm when compared to those less than or equal to 5 cm (hazard ratio = 1.87, *p* = 0.003), probably related to a high risk of an unradical surgery as previously proposed by Ruffini et al. [35,36].

Other revisions have been proposed for the T2 and T3 categories, since significantly better outcomes were reported for patients with a thymoma involving the more easily resectable structures (phrenic nerve or lung) when compared with the invasion of other organs [26,27,28,29,30]. Therefore, unlike the eighth TNM classification, TETs invading the lung or phrenic nerve were downstaged from the T3 category to the T2 category, while the T4 descriptor remained unchanged because of the small number of patients affected by T4 thymomas enrolled in the IASLC’s TD-SPFC database, which consisted mostly of surgical patients [36].

### 3.2. N-Factor

**Figure 1:**
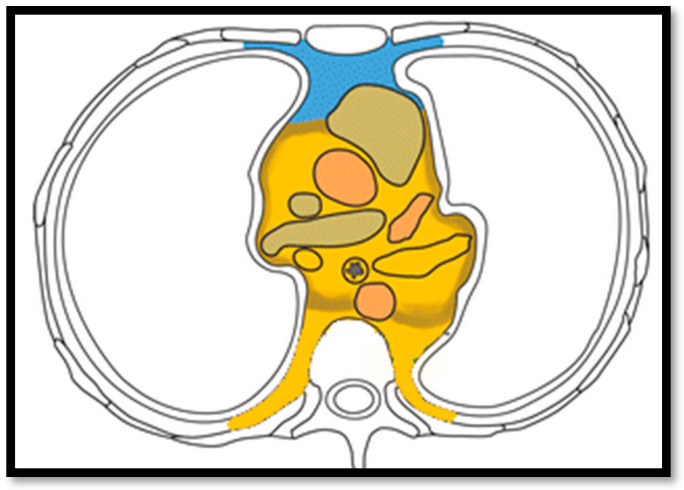
ITMIG/IASLC node compartments for thymic malignancies. Graphic description of N1 (anterior region, blue) and N2 (deep region, yellow) node compartment.

The eighth TNM classification introduced a more detailed nodal involvement description for thymic neoplasm by recognizing, for the first time, three groups according to the occurrence of nodal metastasis and the site of these latter. The absence of nodal metastasis is classified as N0, while N1 and N2 describe the presence of nodal metastases in the anterior compartment (perithymic) and in the deep cervical and thoracic district, respectively (Figure 1). The role of lymphadenectomy in thymic malignancies has always been at the center of debate in the scientific community because nodal involvement could be found in less than 2% of thymoma and in almost 30% of thymic carcinoma [37]. Moreover, while nodal involvement has proven to be a prognostic factor, the role of the number of involved nodes/stations, their location, and the extracapsular extension remain unclear and, even in data collected for a TNM proposal, no statistically significant differences were found in outcome analysis among the N categories [38]. Also, the impact of nodal status on the administration of any adjuvant therapy has never been deeply investigated.

The new upcoming proposal made by the AJCC and the UICC for the ninth edition of the TNM did not introduce any modification for the N and M components. Despite the higher number of enrolled patients with a nodal status assessment, in fact, the authors confirmed the oncological outcomes of N + and M + patients, not requiring other updates compared to the last edition [39].

Weksler and colleagues in 2005 conducted a retrospective study based on the surveillance, epidemiology, and end results (SEER) database, focusing on 442 patients who underwent nodal sampling for thymic malignancies. The authors found a 13.3% of misdiagnosed nodal involvement, of which 23% in clinical early-stage thymoma (namely stage I and II according to Masaoka-Koga) and a doubled risk of death compared with N0 patients; as well, Kondo et al. found a lower rate of 5-year survival in patients with nodal involvement (61.5% vs. 95.6%) [40]. Nowadays, when intraoperative sampling/dissection of nodes is inconsistently practiced, Ruffini et al. published data of an international survey on the impact of the TNM classification in thymic tumors. They reported that lymph node dissection was performed at most in 50% of thymomas and 66% of thymic carcinomas, with clear differences between Asiatic groups and European or American groups [41]. Gu et al., indeed, in a recent retrospective analysis based on the Chinese Alliance for Research in Thymomas (ChART) database, found only 35 cases of lymph node metastases out of 1617 tumor malignancies treated, of which only 7 (0.5%) were in patients with thymoma. The authors confirmed that lymphatic involvement may significantly impact overall survival, but they also added that high histological grade and advanced stages turned out to be independent risk factors predicting lymph node metastasis. Therefore, the assessment of the specific prognostic weight of lymphogenous metastases is a very challenging issue [42].

### 3.3. M-Factor

The M-factor describes the presence of pleural and pericardial nodules (M1a) and lung or distant organ metastases (M1b) and, since nodal involvement is rare, the presence of one or the other results in the IVa or IVb stage according to the TNM classification, similarly to what is described in the Masaoka-Koga staging system. One of the historically most controversial issues is the real impact on global survival of pleural and pericardial implants. In several cases, even a high number of pleuro-pericardial droplets may be easily treated by surgery or multimodal therapy either in stage IVa de novo or in the case of recurrences [30]. Kondo et al., in the analysis of data which led to the eighth TNM edition’s N and M categories, described worse outcomes in terms of survival and recurrence rate for stage IV patients, although this group was underrepresented compared to earlier-stage tumors [43]. Conversely, Ried et al. found a significant better overall survival in stage IV patients with pleural or lymphatic spread than in stage III patients [17]. The lack of randomized studies has not allowed for drawing any strong conclusion on the real prognostic impact of metastatic thymoma. Moreover, in recent decades, also in case of pleuro-pericardial metastastes, different but not standardized multimodal therapeutic strategies have been promoted with interesting results [44]. One of the most interesting multimodal approaches is represented by Hyperthermic Intrathoracic Chemotherapy (HITHOC). This technique synergically combines cytoreductive surgery with macroscopic complete resection, with attention to the oncological effects of hyperthermia and some antineoplastic agents [45]. This technique showed very optimistic results with a 5-year survival up to 75% and excellent local disease control in selected patients [46,47].

## 4. Discussion

Clinical classification is one of the most debated issues regarding the management of thymomas, due to their high heterogeneity and the difficulty of predicting outcomes. The Masaoka-Koga classification has been widely applied for several years thanks to its functionality and practicality, despite its many inaccuracies [10]. Nevertheless, it gained a large consensus because it was designed by surgeons for a disease whose treatment is mainly surgical. With improvements in imaging technologies, growing attention to locally advanced TETs, and the development of new multimodal treatment strategies, the Masaoka-Koga staging system became out-of-date. One of the most critical issues is the scarce role in the clinical staging of early stage TETs, whereas it is not possible to define capsular involvement in pre-operative imaging. Moreover, the Masaoka-Koga stage III is too heterogeneous since it encloses resectable, potentially resectable, and unresectable tumors [13,14]. A similar consideration could be applied to stage IV, which comprehends pathologies with different prognoses which could benefit from a more accurate stratification even in light of nonsurgical treatments [30]. The eighth TNM classification introduced for the first time a new staging system of thymic tumors that represented an inevitable and necessary evolution in order to stratify homogeneous groups of patients with similar features and prognoses. Moreover, one of its most interesting features was its international and multidisciplinary concentration, thanks to data from more than 10,000 patients coming from 105 institutions worldwide. Despite the efforts of several authors and the lack of success of previous systems, this staging system has gained a broad consensus among the scientific community, bringing new attention to several aspects as a more accurate evaluation of primary tumor extent and making clear the need for a detailed nodal dissection even in this kind of tumor, in which scarce attention has been shown to lymphatic and hematogenous dissemination. Nevertheless, some concerns have emerged over time, like the debated prognosis of T1 patients and the real role of the capsule invasion (especially in the analysis of recurrence-free survival); the unclear role of pericardial invasion in the T2 group, which includes both focal and full-thickness infiltration; and the prognosis of the T3 group which, also in this system, incorporates tumors with several infiltration degrees of different organs (some of which are expendable or reconstructable). Some issues, moreover, have been considered merely speculative, such as the distinction between stages IIIa and IIIb, N1 and N2, and M1a and M1b [40]. The upcoming ninth edition of the TNM of TET will fix some of the abovementioned issues and will introduce, for the first time, dimensional criteria [18]. In the light of a surgical resection, whereas minimally invasive surgery is gaining a broader consensus, size matters [48]. Despite several authors having reported complete resection with good oncological outcomes after a minimally invasive resection of large mediastinal masses, the recommended approach for tumors larger than 5 cm or involving great vessels is open surgery [49,50]. The dimensional detail could therefore be helpful in identifying preoperatively the better surgical technique, especially in referred centers with experience in both open and minimally invasive surgery [48]. Lastly, the downstaging from T3 to T2 of thymomas involving lung parenchyma or phrenic nerve demonstrates once again the importance of the surgical perspective in prognostic stratification. Actually, lung and the phrenic nerve could be considered resectable structures since they form a pair, are easily removable, and in most cases don’t need to be replaced or reconstructed. However, it is necessary to bear in mind that about 30% of thymoma patients suffer from Myasthenia Gravis, a severe disease often associated with poor general conditions [51]. In those patients, a more conservative approach to the respiratory organs is advisable, regardless of the stage of disease and even at the price of an unradical resection, in order to preserve functionality [27].

## 5. Conclusions

The TNM staging system represents an essential evolution in the management of such rare tumors and the cornerstone for future research.

## Figures and Tables

**Table 1:** Masaoka-Koga staging system.

Stages	Description
**Stage I**	Grossly and microscopically completely encapsulated tumor
**Stage IIa**	Microscopic transcapsular invasion
**Stage IIb**	Macroscopic invasion into thymic or surrounding fatty tissue, or grossly adherent to but not breaking through mediastinal pleura or pericardium
**Stage III**	Macroscopic invasion into neighboring organ (i.e., pericardium, great vessel, or lung)
**Stage IVa**	Pleural or pericardial metastases
**Stage IVb**	Lymphogenous or hematogenous metastasis

**Table 2:** Eighth staging of thymic epithelial tumors as proposed by IASLC/ITMIG.

Category	Definition *		
**T1a**	Encapsulated or unencapsulated, with or without extension into mediastinal fat		
**T1b**	Extension into mediastinal pleura		
**T2**	Pericardium		
**T3**	Lung, brachiocephalic vein, superior vena cava, chest wall, phrenic nerve, hilar (extrapericardial) pulmonary vessels		
**T4**	Aorta, arch vessels, main pulmonary artery, myocardium, trachea, or esophagus		
**N0**	No nodal involvement		
**N1**	Anterior (perithymic) nodes		
**N2**	Deep intrathoracic or cervical nodes		
**M0**	No metastatic pleural, pericardial, or distant sites		
**M1a**	Separate pleural or pericardial nodule(s)		
**M1b**	Pulmonary intraparenchymal nodule or distant organ metastasis		
**Stage**	**T**	**N**	**M**
**I**	**T1**	N0	M0
**II**	**T2**	N0	M0
**IIIa**	**T3**	N0	M0
**IIIb**	**T4**	N0	M0
**IVa**	T any	**N1**	M0
	T any	N0, 1	**M1a**
**IVb**	T any	**N2**	M0, 1a
	T any	N any	**M1b**

* Definition is involvement of these elements.

**Table 3:** Ninth Proposal for Staging of Thymic Epithelial Tumors.

Parameter	Description
**T1**	Tumor limited to the thymus with or without encapsulation, or directly invades into the mediastinum alone, or directly invades the mediastinal pleura but does not involve any other mediastinal structure.
**T1a**	5 cm or less in its greatest dimension *
**T1b**	larger than 5 cm in its greatest dimension *
**T2**	Tumor directly invades the pericardium (either partial or full thickness), or the lung or the phrenic nerve
**T3**	Tumor directly invades any of the following: (1) brachiocephalic vein, (2) superior vena cava, (3) chest wall or (4) extrapericardial pulmonary arteries or veins
**T4**	Tumor directly invades any of the following: (1) aorta (ascending, arch, or descending), (2) arch vessels, (3) intrapericardial pulmonary artery or veins, (4) myocardium, (5) trachea, or (6) esophagus
**N0**	No nodal involvement
**N1**	Anterior (peri thymic) nodes
**N2**	Deep intrathoracic or cervical nodes (e.g., paratracheal, subcarinal, aortopulmonary window, hilar, jugular, and supraclavicular nodes)
**M0**	No metastatic pleura, pericardial or distant sites
**M1a**	Separate pleural or pericardial nodule(s)
**M1b**	Pulmonary intra-paranchimal nodule or distant organ metastasis

* Irrespective of mediastinal pleura invasion. Mediastinal pleura invasion to be recorded as Additional histologic descriptor.
